# Clinical characteristics of Coronavirus Disease 2019 patients in Beijing, China

**DOI:** 10.1371/journal.pone.0234764

**Published:** 2020-06-17

**Authors:** Zhenhuan Cao, Tongzeng Li, Lianchun Liang, Haibo Wang, Feili Wei, Sha Meng, Miaotian Cai, Yulong Zhang, Hui Xu, Jiaying Zhang, Ronghua Jin

**Affiliations:** 1 Department of Infectious Disease, Beijing You’an Hospital, Capital Medical University, Beijing, China; 2 Peking University Clinical Research Institute, Beijing, China; 3 Beijing Institute of Hepatology, Beijing, China; 4 Department of Science and Technology, Beijing You’an Hospital, Capital Medical University, Beijing, China; Beijing Key Laboratory of Diabetes Prevention and Research, CHINA

## Abstract

The outbreak of Coronavirus Disease (COVID-19) in Wuhan have affected more than 250 countries and regions worldwide. However, most of the clinical studies have been focused on Wuhan, and little is known about the disease outside of Wuhan in China. In this retrospective cohort study, we report the early clinical features of 80 patients with COVID-19 admitted to the hospital in Beijing. The results show that 27 (33.8%) patients had severe illness. Six (7.5%) patients were admitted to the ICU, and 3 (3.8%) patients died. Forty-eight percent (39/80) of the patients had a history of living/traveling in Wuhan. Patients with severe- illness were significantly older (average age, 71 years old vs 44 years old) and had a high incidence of expectoration (59.3% vs 34.0%), shortness of breath (92.6% vs 9.4%), anorexia (51.9% vs 18.9%) and confusion(18.5% vs 0%) compared with nonsevere patients. The systolic blood pressure (median, 130 mmHg vs 120 mmHg) was higher and the oxygen saturation (median, 98.3% vs 92.0%) was significantly lower in severe patients than nonsevere patients. In addition, myoglobin (median, 56.0 ng/mL vs 35.0 ng/mL), troponin I (median, 0.02 pg/mL vs 0.01 pg/mL), C-reactive protein (median, 69.7 mg/L vs 12.9 mg/L) and neutrophils (median, 3.3×10^9^/L vs 2.2×10^9^/L) were significantly increased, while lymphocytes (median, 0.8×10^9^/L vs 1.2×10^9^/L), albumin (mean, 32.8 g/L vs 36.8 g/L) and the creatinine clearance rate (median, 91.2 vs 108.2 ml/min/1.73m^2^) were significantly decreased among severe patients. Our study revealed that older patients with high levels of C-reactive protein, myoglobin, troponin I, and neutrophil and high systolic blood pressure as well as low levels of lymphocytes, and albumin and a low creatinine clearance rate and oxygen saturation were more likely to have severe disease.

## Introduction

The outbreak of Coronavirus Disease (COVID-19) from Wuhan, China, has affected more than 250 countries and regions worldwide in only two months [1, 2]. As of April 1, 2020, the number of confirmed cases worldwide has reached 911308, and the number of deaths is 45497 [3]. From the existing epidemiological data, it can be seen that the epidemic caused by severe acute respiratory syndrome coronavirus 2 (SARS-CoV-2) is far more widespread and contagious than that caused by severe acute respiratory syndrome coronavirus (SARS-CoV) and Middle East respiratory syndrome coronavirus (MERS-CoV) [4, 5].The sudden appearance of this infectious disease has become the most serious problem affecting public health and social and economic development at present [6].

To date, most clinical studies on COVID-19 have focused on describing the general epidemiologic and clinical characteristics in Wuhan [7–9]. However, little is known about these characteristics outside of Wuhan. In this paper, we report the early clinical features of 80 patients with COVID-19 admitted to the hospital in Beijing. This will not only identify the defining clinical characteristics in different places, but also reveal the risk factors associated with severe illness.

## Materials and methods

### Study design

This study was a single- center retrospective cohort study. We enrolled all patients with confirmed SARS-CoV-2 infection hospitalized at Beijing You’an Hospital from Jan 21 to Feb 12, 2020, in Beijing, China. Clinical data were obtained from electronic medical records, including demographic data, exposure history, signs and symptoms, and laboratory data at admission. The final date of follow-up was February 18, 2020.

### Patients

All patients with COVID-19 enrolled in this study were diagnosed according to the guidelines for the diagnosis and treatment of pneumonia due to infection with the novel coronavirus (Trial 5th Edition, in Chinese) [10] after the exclusion of influenza A virus and influenza B virus coinfection. All patients were laboratory-confirmed to have SARS-CoV-2 infection (the SARS-CoV-2- specific real-time RT-PCR result was positive). The diagnosis of severe patients was made according to any of the following criteria: 1) respiratory distress indicated by a number of breaths >30 times/min; 2) resting state, oxygen saturation < 93%; 3) arterial partial pressure of oxygen/oxygen concentration <300 mmHg; 4) respiratory failure and a need for mechanical ventilation; 5) shock; and 6) other organ failure requiring intensive care unit (ICU) monitoring and treatment. We grouped patients into severe and nonsevere groups. Severe patients were defined as either those with a severe case on admission or those with a nonsevere case that became severe after admission.

### Clinical data collection

The medical records of patients were analyzed by the research team of Peking University Clinical Research Institute. Clinical data were obtained with data collection forms from electronic medical records. The data were reviewed by a team of physicians (M Cai, Y Zhang, H Xu MM, and J Zhang). Collected information included demographic data (gender, age), exposure history (Wuhan exposure, non-Wuhan exposure), signs and symptoms (fever, maximum temperature, cough, expectoration, shortness of breath, fatigue, anorexia, muscle aches, headache, chills, nausea and vomiting, diarrhea, and confusion, etc.), medical history (hypertension, heart disease, diabetes, history of surgery, chronic obstructive pulmonary disease, liver disease, etc.), smoking history, and treatment measures (i.e., antiviral therapy, corticosteroid therapy, or Chinese medicine). The laboratory data were collected on the admission day, including the results of routine blood tests (white blood cell count, neutrophil count, lymphocyte count, hemoglobin, and platelet count), liver and kidney function (alanine aminotransferase, aspartate aminotransferase, total bilirubin, albumin, creatinine, creatinine clearance rate, etc.), coagulation (prothrombin time, prothrombin activity, and activated partial thromboplastin time), myocardial enzymes (creatine kinase, troponin I, and myochrome), lactic acid, procalcitonin, c-reactive protein, and chest computed tomographic (CT) scans. The date of disease onset was defined as the day when symptoms were first noticed.

### Laboratory testing for SARS-CoV-2

The throat swab samples from patients suspected of having SARS-Cov-2 infection were immediately placed into the collection tube and transferred to a laboratory with in the Biosafety Level 2 Plus facilities at Beijing You’an Hospital, Capital Medical University. The extraction reagent was obtained from a nucleic acid extraction and purification kit based on the magnetic bead method (Shanghai Bio Germmedical Technology Co Ltd). The samples were inactivated at 56°C for 30 minutes and centrifuged at 2000 rpm for 1 minute. Two hundred microliters of sample were obtained for RNA extraction according to the manufacturer's protocol, and then the RNA was eluted in 100 μl elution buffer. The real-time RT-PCR assay was performed using a SARS-Cov-2nucleic acid detection kit (Shanghai Bio Germmedical Technology Co Ltd).

### Statistical analysis

Statistical analyses were performed using SPSS Version 24. All continuous data are presented as the mean ± standard deviation (SD) or median ± interquartile range (IQR). Categorical data are presented as numbers and percentages. Fisher’s exact text or chi-square tests were conducted for the analysis of the categorical variables, and t-tests or Mann-Whitney U tests were conducted for the analysis of the continuous variables to compare the differences between variables and the severity of illness. The significance level was set at *P*<0.05 for all statistical analyses.

### Ethics approval

This study protocol was approved by the institutional ethics board of Beijing You’an Hospital, Capital Medical University (No. [2020]021). Ethics committee waived the requirement for informed consent.

## Results

### Clinical characteristics

From January 21 to February 12, 2020, a total of 80 patients were hospitalized in Beijing You’an Hospital. The patients were divided into severe patients (n = 27, including 15 patients with severe cases on admission and 12 patients with nonsevere cases that became severe after admission) and nonsevere patients (n = 53, who remained nonsevere). Among them, 6 (7.5%) patients were admitted to the ICU, 3 (3.8%) patients died, and 47 (58.8%) patients were discharged by February 18, 2020.

The average age was 53 years old, and 38 of the 80 patients (47.5%) were male. The median time from symptom onset to hospital admission was 3.5 days (IQR range, 2–6), and the median time to the diagnosis of severe illness was 7 days (IQR range, 3–7). Forty-eight percent of the patients had a history of living/traveling in Wuhan. The most common chronic medical illnesses included hypertension (25.0%), cardiovascular disease (12.5%), diabetes (7.5%) and chronic obstructive pulmonary disease (6.3%). Only 5 of 80 patients had a history of smoking. The most common symptom of patients with COVID-19 was fever (86.3%), which was followed by cough (71.3%), expectoration (42.5%), shortness of breath (37.5%), fatigue (37.5%) and anorexia (30.0%). The treatments included antiviral therapy (16.3%, lopinavir and ritonavir tablets or chloroquine diphosphate), corticosteroid therapy (23.8%), Chinese medicine (61.3%, Lianhuaqingwen or Jinhuaqinggan) or a combination of two or three of these drugs. (Table 1)

**Table 1:** Characteristics of patients with COVID-19.

Characteristic	Total (n = 80)	Severe (n = 27)	Nonsevere (n = 53)	*P* value
Sex (M, %)	38(47.5%)	16(59.3)	22(41.5%)	0.16
Age, Mean ± SD, year	53±20	71±15	44±16	<0.01
Wuhan exposure history (n %)	39 (48.8%)	11 (40.7%)	28 (52.8%)	0.35
Signs and symptoms (n %)				
Fever	69(86.3%)	25(92.6%)	44(83.0%)	0.32
Cough	57(71.3%)	22(81.5%)	35(66.0%)	0.20
Expectoration	34(42.5%)	16(59.3%)	18(34.0%)	0.04
Shortness of breath	30(37.5%)	25(92.6%)	5(9.4%)	<0.01
Fatigue	30(37.5%)	13(48.1%)	17(32.1%)	0.22
Anorexia	24(30.0%)	14(51.9%)	10(18.9%)	<0.01
Muscle aches	12(15.0%)	1 (3.7%)	11(20.8%)	0.05
Headache	8(10.0%)	2(7.4%)	6(11.3%)	0.47
Chills	8(10.0%)	1(3.7%)	7(13.2%)	0.26
Nausea and vomiting	6(7.5%)	4(14.8%)	2(3.8%)	0.17
Diarrhea	5(6.3%)	0(0%)	5(9.4%)	0.16
Confusion	5(6.3%)	5(18.5%)	0(0%)	<0.01
Time from the onset of symptoms to admission, median (IQR), d	3.5 (2, 6)	4(2, 6)	3(2, 5.5)	0.29
Time from the onset of symptoms to the diagnosis of severe illness, median (IQR), d	-	7 (3,7)	-	-
Chronic medical illness (n %)				
Hypertension	20(25.0%)	4(14.8%)	16(%)	0.18
Cardiovascular	10(12.5%)	5(18.5%)	5(30.2%)	0.29
Diabetes	6(7.5%)	3(11.1%)	3(5.7%)	0.40
History of surgery	6(7.5%)	0(0%)	6(11.3%)	0.09
Chronic obstructive pulmonary disease	5(6.3%)	0(0%)	5(30.2%)	0.16
Smoking history (n %)	5(6.3%)	4(14.8%)	1(18.9%)	0.04
Systolic blood pressure, median (IQR), mmHg	120(116, 130)	130(120, 140)	120(111, 126)	0.01
Respiratory rate, median (IQR), bpm	20 (20, 21)	21 (20, 22)	20 (20, 20)	<0.01
Pulse, median (IQR), bpm	82 (80, 92)	90 (80, 100)	82 (80, 89)	0.07
Oxygen saturation, median (IQR), %	96.0 (94.0, 99.0)	92.0 (88.0, 94.0)	98.3(96.0, 100.0)	<0.01
Pneumonia manifestations on CT	78(97.5%)	27(100%)	51(96.2%)	0.55
Treatment measures (n %)				
Antiviral therapy	13(16.3%)	3(11.1%)	10(18.9%)	
Corticosteroid therapy	19(23.8%)	14(51.9%)	5(9.4%)	
Chinese medicine	49(61.3%)	14(51.9%)	35 (66.0%)	

We found that patients with severe- illness were significantly older than nonsevere illness and the proportion of male patients was slightly higher. The incidence of the following symptoms in the severe- illness group was significantly increased: expectoration, shortness of breath, anorexia and confusion. We also found that systolic pressure and respiratory rate were higher and oxygen saturation was significantly lower in the severe patients (Table 1). There were no significant differences in chronic medical illness, exposure history, or the time between the onset of symptoms and admission between the two groups.

The three deaths included two males and one female, aged 82 to 94 years old. All three patients had serious chronic medical illnesses, including cardiovascular disease and hypertension. The time from onset to death for the three patients were 7 days, 10 days and 4 days.

### Laboratory parameters in severe and nonsevere patients

There were numerous differences in the laboratory findings between severe and nonsevere patients (Table 2). The total white blood cell count was basically with in the normal range in patients with COVID-19, but lymphocytes were decreased, especially in severe patients. The mean albumin level was as low as 35.4 g/L in patients with COVID-19, and it was significantly lower in severe patients. There was no significant difference in the indicators of liver function injury (alanine aminotransferase, aspartate aminotransferase, and total bilirubin) between the two groups. The renal function of severe patients was significantly worse, which was indicated by the level of creatinine being significantly higher and the creatinine clearance rate being significantly lower. In addition, the levels of myoglobin and troponin I in the severe patients were significantly higher, as well as the levels of lactate and C-reactive protein.

**Table 2:** Laboratory findings of patients with COVID-19 on the admission day.

	Normal Range	Total (n = 80)	Severe (n = 27)	Nonsevere (n = 53)	*P* value
white blood cell count, median (IQR), ×10^9^/L	3.5–9.5	4.0 (3.5, 5.6)	4.9(3.5, 6.5)	3.9(3.4, 4.8)	0.03
neutrophil count, median (IQR), ×10^9^/L	1.8–6.3	2.4 (1.8, 3.5)	3.3 (1.9, 5.5)	2.2 (1.7, 2.8)	<0.01
lymphocyte count, median (IQR), ×10^9^/L	1.1–3.2	1.0 (0.7,1.4)	0.8 (0.6, 0.9)	1.2 (0.8, 1.6)	<0.01
hemoglobin, median (IQR), g/L	130–175	135(124.2,144.8)	135.0 (121.0, 142.0)	135.0(125.5, 145.0)	0.33
platelet count, median (IQR), ×10^9^/L	125–350	189.5(148.3, 242.0)	184 (146.0, 225.0)	190(150.5, 257)	0.39
plateletcrit, median (IQR), %	0.2–0.5	0.2 (0.1,0.2)	0.2 (0.1, 0.2)	0.2(0.1, 0.2)	0.67
alanine aminotransferase, median (IQR),U/L	9–50	28.0(20.0,46.8)	28.0(20.0, 55.0)	28.0(19.5, 45.5)	0.51
aspartate aminotransferase, median (IQR),U/L	15–40	30.0(22.0, 47.0)	32.0(26.0, 54.0)	29.0(19.5, 42.0)	0.09
total bilirubin, median (IQR),μmol /L	5–21	8.6(6.3, 12.1)	10.5(6.7, 13.0)	8.5(6.0, 11.3)	0.25
albumin, Mean±SD, g/L	40–55	35.4±5.5	32.8±6.5	36.8±4.3	0.01
albumin/globulin, Mean±SD, %	1.2–2.4	0.98±0.25	0.8±0.2	1.1±0.2	<0.01
creatinine, median (IQR),μmol/L	57–111	64.5(53.0, 80.5)	78.0(55.0, 91.0)	61.0 (51.5, 73.5)	0.02
creatinine clearance rate, median(IQR),ml/min/1.73m^2^	>90	97.0(81.8, 112.7)	91.2 (63.2,94.9)	108.2(94.2, 118.8)	<0.01
K^+^, Mean±SD, mmol/L	3.5–5.3	3.8±0.5	3.8±0.5	3.7±0.4	0.30
Na^+^, Mean±SD, mmol/L	137–147	135.6±4.5	134.7±7.1	136.0±2.2	0.24
Cl^-^, Mean ± SD, mmol/L	99–110	100.8±6.2	99.3±6.6	101.5±5.8	0.13
Prothrombin time median (IQR), S	9.9–12.8	12.7(11.9, 13.1)	12.7(11.8, 13.4)	12.7(12.2, 13.1)	0.66
prothrombin activity, median (IQR), %	80–120	74.0(71.0, 80.8)	74.0(69.0, 83.0)	74.0(71.0, 79.0)	0.73
Activated partial thromboplastin time, median (IQR), S	25–36.5	32.8(30.5, 34.9)	32.0(28.5, 34.2)	33.5(30.8, 35.4)	0.04
creatine kinase, median (IQR), U/L	50–310	81.0(46.0, 137.0)	89.0(53.0 195.0)	69.0(45.0, 117.5)	0.17
myochrome, median (IQR), ng/mL	16–96	43.5(29.3, 72.5)	56.0(45.0, 159.0)	35.0(28.0, 57.0)	<0.01
troponin I, median (IQR), pg/mL	<0.056	0.01(0.01,0.02)	0.02(0.01, 0.07)	0.01(0.01, 0.01)	<0.01
lactate, Mean±SD, mmol/L	0.4–2.0	1.3±0.6	1.5±0.7	1.2±0.5	0.04
C-reactive protein, median (IQR), mg/L	<3	18.7(5.6, 58.2)	69.7(19.3, 111.6)	12.9(2.5, 23.5)	<0.01
procalcitonin, median (IQR), ng/mL	<0.1	0.11(0.10, 0.14)	0.1(0.1, 0.2)	0.1(0.1, 0.1)	0.06

## Discussion

COVID-19 is an infectious disease, with a ferocious course that has infected a large number of people [11]. The clinical manifestations of patients are variable and the disease outcomes are also very different [12]. Beijing You’an Hospital was assigned by the government to treat COVID-19 patients in Beijing, so our data can partially represent SARS-CoV-2 infection in Beijing.

In our study, a total of 80 patients hospitalized in Beijing You’an Hospital from January 21 to February 12, 2020 were retrospectively analyzed. The incidence of severe illness was 33.8% (27/80). The median time from the onset of symptoms to admission was 3.5 days (IQR range, 2–6) and the median time to the diagnosis of severe illness was 7 days (IQR range, 3–7). Only 6 (7.5%) patients were admitted to the ICU, and 3 (3.8%) patients died, which represents a death rate that is lower than the rate reported in previous studies. Huang C et al [9] reported that 32% of patients needed ICU treatment and Wang D et al [8] reported that 26% needed ICU treatment. The reason may be that our study was conducted in Beijing, and only 48.8% of patients had Wuhan exposure, which is consistent with the report by Chang D [13].

All of the patients in this study received supportive treatments. Most patients (61.3%) received Chinese medicine. Some seriously ill patients were treated with antiviral drugs and glucocorticoids. The doses of glucocorticoids and antiviral drugs used varied depending on the disease severity. To date, no specific treatment has been recommended for coronavirus infection except for meticulous supportive therapy [14].

We found that patients with severe- illness were older and were more likely to be males, which is consistent with previous studies. The number of smokers was relatively small, and there were only 5 (6.25%) cases involving smokers in this study. The most common chronic medical illnesses included hypertension (25%), cardiovascular disease (12.5%), diabetes (7.5%) and chronic obstructive pulmonary disease (6.3%). There were no differences between the two groups regarding chronic medical illness. The most common symptoms of COVID-19 were fever (86.3%), cough (71.3%), expectoration (42.5%), and shortness of breath (37.5%). We found that patients with severe-illness were more likely to have expectoration and shortness of breath. The systolic blood pressure and respiratory rate were higher, and the oxygen saturation was lower in patients with severe- illness.

There were numerous differences in laboratory findings between the two groups. We found that lymphocytes were significantly reduced in patients with COVID-19, especially in patients with severe-illness. N Chen [7] reported that the levels of alanine aminotransferase and aspartate aminotransferase which represent liver damage were elevated in 28–35% of patients with COVID-19. Unlike the results of this study, the levels of alanine aminotransferase and aspartate aminotransferase were normal in our study. We found that the albumin level was generally decreased in COVID-19 patients and was significantly decreased in patients with severe- illness. In addition, the levels of C-reactive protein, myochrome, troponin I, and lactate were significantly higher in severe patients.

This study has several limitations. First, respiratory tract specimens were used to diagnose COVID-19 through RT-PCR. The serum of patients was not obtained to evaluate the viremia. Second, among the 80 cases in this study, some patients are still hospitalized at the time of manuscript submission, and continued observations of their disease are needed.

## Supporting information

S1 Data (XLSX)
