# Supplementary material for: Clinical characteristics of Coronavirus Disease 2019 patients in Beijing, China
Source: PLoS One. 2020 Jun 17;15(6):e0234764. doi: 10.1371/journal.pone.0234764 (PMC7299347; doi:10.1371/journal.pone.0234764)
Supplement: Attachment — Submitted filename: Caio et al. PLOS.In this manuscript.docx [file pone.0234764.s002.docx]

In this manuscript, Cao et al described Epidemiologic and clinical characteristics of COVID-19 infected patients in Beijing, China. This is a straightforward descriptive paper. Given the importance of COVID 19 infection in China, the paper add some important information outside Wuhan, which is the epicenter of the epidemics. However, the paper lacks very important information, specifically

1. There was no comparison of the Epidemiologic and clinical characteristics of COVID-19 infected patients in Beijing with those in Wuhan.

2. What kind of antiviral and Chinese medicines were used in treatment of Beijing patients?

3. Since the data were collected at the time of admission, it is not clear whether patients already showed severity of symptoms. How many of non-severe patients showed severe symptoms later on while in the hospital?

4. In Discussion it is mentioned, “Patients with severe illness were more likely to have expectoration and shortness of breath, however in most cases severity was defined by these parameters. Therefore, severity must be better defined.

5. Minor point: spelling must be checked. Beijing in title is misspelled.
