# Supplementary material for: Clinical characteristics of Coronavirus Disease 2019 patients in Beijing, China
Source: PLoS One. 2020 Jun 17;15(6):e0234764. doi: 10.1371/journal.pone.0234764 (PMC7299347; doi:10.1371/journal.pone.0234764)
Supplement: Attachment — Submitted filename: Response to Reviewers.docx [file pone.0234764.s003.docx]

\# academic editor

1. The language should be edited extensively for better quality.

**R: The** **manuscript was edited for proper English language, grammar, punctuation, spelling, and overall style by one or more of the highly qualified native English speaking editors at AJE.**

2. It is inappropriate to state "Epidemiologic" in the title because the data were obtained only from one hospital.

**R: The reviewer gave us constructive suggestions and we agree on this comment. "Epidemiologic" was removed from the text.**

3."Corona Virus Disease" in the title of the paper may be confused with other coronavirus disease. It may be better using "coronavirus disease 2019" whose abbreviation is COVID-19 according to the WHO nomenclature.

**R：We revised the article in accordance with your comments.**

4. Statistical analysis for continuous variables should be Mann-Whitney U when the data show non-normal distribution rather than t test.

**R: In the revised version, the detail of statistical analysis for continuous variables were included and highlighted in the text.**

**Statistical analyses were performed using SPSS Version 24. All continuous data were present as mean ± standard deviation (SD) or median ± interquartile range (IQR). Categorical data were presented as number and percentages. Fisher’s exact or chi-square tests were conducted for the categorical variables, and t-test or Mann-Whitney U were conducted for continuous variables, respectively, to compare the difference between variables and the severe illness. The significance level was set at P<0.05 for all statistical analyses.**

5. There is no outcomes of the dead patients.

**R: The detail information of dead patients was added and shown in the text (page 8, line 164-167).**

**The three deaths included two males and one female, aged 82 to 94 years old. All three patients had serious chronic medical illnesses, including cardiovascular disease and hypertension. The time from onset to death for the three patients were 7 days, 10 days and 4 days.**

#reviewer 1
In this manuscript, Cao et al described Epidemiologic and clinical characteristics of COVID-19 infected patients in Beijing, China. This is a straightforward descriptive paper. Given the importance of COVID 19 infection in China, the paper add some important information outside Wuhan, which is the epicenter of the epidemics. However, the paper lacks very important information, specifically

1. There was no comparison of the Epidemiologic and clinical characteristics of COVID-19 infected patients in Beijing with those in Wuhan.

**R: This manuscript was aimed to describe the clinical characteristics of COVID-19 infected patients in Beijing. We have no data of patients in Wuhan. In discussion, we made a relevant comparison with previous reports in Wuhan.**

2. What kind of antiviral and Chinese medicines were used in treatment of Beijing patients?

**R: Antiviral medicines included lopinavir and ritonavir tablets and chloroquine diphosphate. Chinese medicine included Lianhuaqingwen and Jinhuaqinggan.**

**This information was supplemented in the manuscript and was shown on pages 7, line 151-153.**

3. Since the data were collected at the time of admission, it is not clear whether patients already showed severity of symptoms. How many of non-severe patients showed severe symptoms later on while in the hospital?

**R: The patients were divided into severe patients (n=27, including 15 patients with severe cases on admission and 12 patients with nonsevere cases that became severe after admission)** **and nonsevere patients (n=53, who remained nonsevere).**

**This result is shown in result section, in page 7, line 138. I highlighted it in the manuscript.**

4. In Discussion it is mentioned, “Patients with severe illness were more likely to have expectoration and shortness of breath, however in most cases severity was defined by these parameters. Therefore, severity must be better defined.

**R: The reviewer gave us constructive suggestions and we agree on this comment. The diagnosis of severe patients was made according to the guidelines for the diagnosis and treatment of pneumonia due to infection with the novel coronavirus (Trial 5th Edition, in Chinese)**

5. Minor point: spelling must be checked. Beijing in title is misspelled

**R: I have made a correction in the article. The manuscript was edited for proper English language, grammar, punctuation, spelling, and overall style by one or more of the highly qualified native English speaking editors at AJE.**
