# Supplementary material for: Clinical characteristics of Coronavirus Disease 2019 patients in Beijing, China
Source: PLoS One. 2020 Jun 17;15(6):e0234764. doi: 10.1371/journal.pone.0234764 (PMC7299347; doi:10.1371/journal.pone.0234764)
Supplement: Peer review history

# Peer review: Decision Letter 0

**DOI:** 10.1371/journal.pone.0234764.r001

**Authors:** Ying-Mei Feng

**Transfer Alert**

This paper was transferred from another journal. As a result, its full editorial history (including decision letters, peer reviews and author responses) may not be present.

22 Apr 2020

PONE-D-20-08032

Epidemiologic and Clinical Characteristics of Corona Virus Disease Involving Patients in Beijng, China

PLOS ONE

Dear Dr. Cao,

Thank you for submitting your manuscript to PLOS ONE. After careful consideration, we feel that it has merit but does not fully meet PLOS ONE’s publication criteria as it currently stands. Therefore, we invite you to submit a revised version of the manuscript that addresses the points raised during the review process.

# academic editor

1. The language should be edited extensively for better quality.

2. It is inappropriate to state "Epidemiologic" in the title because the data were obtained only from one hospital.

3."Corona Virus Disease" in the title of the paper may be confused with other coronavirus disease. It may be better using "coronavirus disease 2019" whose abbreviation is COVID-19 according to the WHO nomenclature.

4. Statistical analysis for continuous variables should be Mann-Whitney U when the data show non-normal distribution rather than t test.

5. There is no outcomes of the dead patients. 

#reviewer 1

In this manuscript, Cao et al described Epidemiologic and clinical characteristics of COVID-19 infected patients in Beijing, China. This is a straightforward descriptive paper. Given the importance of COVID 19 infection in China, the paper add some important information outside Wuhan, which is the epicenter of the epidemics. However, the paper lacks very important information, specifically

1. There was no comparison of the Epidemiologic and clinical characteristics of COVID-19 infected patients in Beijing with those in Wuhan.

2. What kind of antiviral and Chinese medicines were used in treatment of Beijing patients?

3. Since the data were collected at the time of admission, it is not clear whether patients already showed severity of symptoms. How many of non-severe patients showed severe symptoms later on while in the hospital?

4. In Discussion it is mentioned, “Patients with severe illness were more likely to have expectoration and shortness of breath, however in most cases severity was defined by these parameters. Therefore, severity must be better defined.

5. Minor point: spelling must be checked. Beijing in title is misspelled

We would appreciate receiving your revised manuscript by Jun 06 2020 11:59PM. When you are ready to submit your revision, log on to https://www.editorialmanager.com/pone/ and select the 'Submissions Needing Revision' folder to locate your manuscript file.

If you would like to make changes to your financial disclosure, please include your updated statement in your cover letter.

To enhance the reproducibility of your results, we recommend that if applicable you deposit your laboratory protocols in protocols.io, where a protocol can be assigned its own identifier (DOI) such that it can be cited independently in the future. For instructions see: http://journals.plos.org/plosone/s/submission-guidelines#loc-laboratory-protocols

Please include the following items when submitting your revised manuscript:

- A rebuttal letter that responds to each point raised by the academic editor and reviewer(s). This letter should be uploaded as separate file and labeled 'Response to Reviewers'.
- A marked-up copy of your manuscript that highlights changes made to the original version. This file should be uploaded as separate file and labeled 'Revised Manuscript with Track Changes'.
- An unmarked version of your revised paper without tracked changes. This file should be uploaded as separate file and labeled 'Manuscript'.

Please note while forming your response, if your article is accepted, you may have the opportunity to make the peer review history publicly available. The record will include editor decision letters (with reviews) and your responses to reviewer comments. If eligible, we will contact you to opt in or out.

We look forward to receiving your revised manuscript.

Kind regards,

Ying-Mei Feng

Academic Editor

PLOS ONE

Journal requirements:

When submitting your revision, we need you to address these additional requirements.

1. Please ensure that your manuscript meets PLOS ONE's style requirements, including those for file naming. The PLOS ONE style templates can be found at https://journals.plos.org/plosone/s/file?id=wjVg/PLOSOne_formatting_sample_main_body.pdf and https://journals.plos.org/plosone/s/file?id=ba62/PLOSOne_formatting_sample_title_authors_affiliations.pdf

2. In ethics statement in the manuscript and in the online submission form, please provide additional information about the patient records/samples used in your retrospective study. Specifically, please ensure that you have discussed whether all data/samples were fully anonymized before you accessed them and/or whether the IRB or ethics committee waived the requirement for informed consent. If patients provided informed written consent to have data/samples from their medical records used in research, please include this information

3. We suggest you thoroughly copyedit your manuscript for language usage, spelling, and grammar. If you do not know anyone who can help you do this, you may wish to consider employing a professional scientific editing service.  

Whilst you may use any professional scientific editing service of your choice, PLOS has partnered with both American Journal Experts (AJE) and Editage to provide discounted services to PLOS authors. Both organizations have experience helping authors meet PLOS guidelines and can provide language editing, translation, manuscript formatting, and figure formatting to ensure your manuscript meets our submission guidelines. To take advantage of our partnership with AJE, visit the AJE website (http://learn.aje.com/plos/) for a 15% discount off AJE services. To take advantage of our partnership with Editage, visit the Editage website (www.editage.com) and enter referral code PLOSEDIT for a 15% discount off Editage services.  If the PLOS editorial team finds any language issues in text that either AJE or Editage has edited, the service provider will re-edit the text for free.

Upon resubmission, please provide the following:

- The name of the colleague or the details of the professional service that edited your manuscript
- A copy of your manuscript showing your changes by either highlighting them or using track changes (uploaded as a *supporting information* file)

4. We note that you have stated that you will provide repository information for your data at acceptance. Should your manuscript be accepted for publication, we will hold it until you provide the relevant accession numbers or DOIs necessary to access your data. If you wish to make changes to your Data Availability statement, please describe these changes in your cover letter and we will update your Data Availability statement to reflect the information you provide.

5. Thank you for stating the following financial disclosure:

'NO - Include this sentence at the end of your statement: The funders had no role in

study design, data collection and analysis, decision to publish, or preparation of the

manuscript.'

At this time, please address the following queries:

1. Please clarify the sources of funding (financial or material support) for your study. List the grants or organizations that supported your study, including funding received from your institution.
2. State what role the funders took in the study. If the funders had no role in your study, please state: “The funders had no role in study design, data collection and analysis, decision to publish, or preparation of the manuscript.”
3. If any authors received a salary from any of your funders, please state which authors and which funders.
4. If you did not receive any funding for this study, please state: “The authors received no specific funding for this work.”

Please include your amended statements within your cover letter; we will change the online submission form on your behalf.

Attachment Submitted filename: Caio et al. PLOS.In this manuscript.docx Click here for additional data file.

# Author comment: Author response to Decision Letter 0

**DOI:** 10.1371/journal.pone.0234764.r002

18 May 2020

# academic editor

1. The language should be edited extensively for better quality.

R: The manuscript was edited for proper English language, grammar, punctuation, spelling, and overall style by one or more of the highly qualified native English speaking editors at AJE.

2. It is inappropriate to state "Epidemiologic" in the title because the data were obtained only from one hospital.

R: The reviewer gave us constructive suggestions and we agree on this comment. "Epidemiologic" was removed from the text.

3."Corona Virus Disease" in the title of the paper may be confused with other coronavirus disease. It may be better using "coronavirus disease 2019" whose abbreviation is COVID-19 according to the WHO nomenclature.

R：We revised the article in accordance with your comments.

4. Statistical analysis for continuous variables should be Mann-Whitney U when the data show non-normal distribution rather than t test.

R: In the revised version, the detail of statistical analysis for continuous variables were included and highlighted in the text.

Statistical analyses were performed using SPSS Version 24. All continuous data were present as mean ± standard deviation (SD) or median ± interquartile range (IQR). Categorical data were presented as number and percentages. Fisher’s exact or chi-square tests were conducted for the categorical variables, and t-test or Mann-Whitney U were conducted for continuous variables, respectively, to compare the difference between variables and the severe illness. The significance level was set at P<0.05 for all statistical analyses.

5. There is no outcomes of the dead patients. 

R: The detail information of dead patients was added and shown in the text (page 8, line 164-167).

The three deaths included two males and one female, aged 82 to 94 years old. All three patients had serious chronic medical illnesses, including cardiovascular disease and hypertension. The time from onset to death for the three patients were 7 days, 10 days and 4 days.

#reviewer 1

1. There was no comparison of the Epidemiologic and clinical characteristics of COVID-19 infected patients in Beijing with those in Wuhan.

R: This manuscript was aimed to describe the clinical characteristics of COVID-19 infected patients in Beijing. We have no data of patients in Wuhan. In discussion, we made a relevant comparison with previous reports in Wuhan.

2. What kind of antiviral and Chinese medicines were used in treatment of Beijing patients?

R: Antiviral medicines included lopinavir and ritonavir tablets and chloroquine diphosphate. Chinese medicine included Lianhuaqingwen and Jinhuaqinggan. 

This information was supplemented in the manuscript and was shown on pages 7, line 151-153.

3. Since the data were collected at the time of admission, it is not clear whether patients already showed severity of symptoms. How many of non-severe patients showed severe symptoms later on while in the hospital?

R: The patients were divided into severe patients (n=27, including 15 patients with severe cases on admission and 12 patients with nonsevere cases that became severe after admission) and nonsevere patients (n=53, who remained nonsevere).

This result is shown in result section, in page 7, line 138. I highlighted it in the manuscript.

4. In Discussion it is mentioned, “Patients with severe illness were more likely to have expectoration and shortness of breath, however in most cases severity was defined by these parameters. Therefore, severity must be better defined.

R: The reviewer gave us constructive suggestions and we agree on this comment. The diagnosis of severe patients was made according to the guidelines for the diagnosis and treatment of pneumonia due to infection with the novel coronavirus (Trial 5th Edition, in Chinese)

5. Minor point: spelling must be checked. Beijing in title is misspelled

R: I have made a correction in the article. The manuscript was edited for proper English language, grammar, punctuation, spelling, and overall style by one or more of the highly qualified native English speaking editors at AJE.

Attachment Submitted filename: Response to Reviewers.docx Click here for additional data file.

# Editor report: Decision Letter 1

**DOI:** 10.1371/journal.pone.0234764.r003

**Authors:** Ying-Mei Feng

3 Jun 2020

Clinical Characteristics of Coronavirus Disease 2019 Patients in Beijing, China

PONE-D-20-08032R1

Dear Dr. Jin,

We’re pleased to inform you that your manuscript has been judged scientifically suitable for publication and will be formally accepted for publication once it meets all outstanding technical requirements.

Within one week, you’ll receive an e-mail detailing the required amendments. When these have been addressed, you’ll receive a formal acceptance letter and your manuscript will be scheduled for publication.

An invoice for payment will follow shortly after the formal acceptance. To ensure an efficient process, please log into Editorial Manager at http://www.editorialmanager.com/pone/, click the 'Update My Information' link at the top of the page, and double check that your user information is up-to-date. If you have any billing related questions, please contact our Author Billing department directly at authorbilling@plos.org.

If your institution or institutions have a press office, please notify them about your upcoming paper to help maximize its impact. If they’ll be preparing press materials, please inform our press team as soon as possible -- no later than 48 hours after receiving the formal acceptance. Your manuscript will remain under strict press embargo until 2 pm Eastern Time on the date of publication. For more information, please contact onepress@plos.org.

Kind regards,

Ying-Mei Feng

Academic Editor

PLOS ONE

# Editor report: Acceptance letter

**DOI:** 10.1371/journal.pone.0234764.r004

**Authors:** Ying-Mei Feng

8 Jun 2020

PONE-D-20-08032R1 

Clinical Characteristics of Coronavirus Disease 2019 Patients in Beijing, China 

Dear Dr. Jin:

I'm pleased to inform you that your manuscript has been deemed suitable for publication in PLOS ONE. Congratulations! Your manuscript is now with our production department. 

If your institution or institutions have a press office, please let them know about your upcoming paper now to help maximize its impact. If they'll be preparing press materials, please inform our press team within the next 48 hours. Your manuscript will remain under strict press embargo until 2 pm Eastern Time on the date of publication. For more information please contact onepress@plos.org.

If we can help with anything else, please email us at plosone@plos.org. 

Thank you for submitting your work to PLOS ONE and supporting open access. 

Kind regards, 

PLOS ONE Editorial Office Staff

on behalf of

Dr Ying-Mei Feng 

Academic Editor

PLOS ONE
